# SVep1, a temperate phage of human oral commensal *Streptococcus vestibularis*

**DOI:** 10.3389/fmicb.2023.1256669

**Published:** 2023-09-12

**Authors:** Miaomiao Wu, Yanpeng Zhu, Yuhui Yang, Yali Gong, Zongyue Chen, Binyou Liao, Yu Xiong, Xia Zhou, Yan Li

**Affiliations:** ^1^State Key Laboratory of Oral Diseases and National Center for Stomatology and National Clinical Research Center for Oral Diseases, West China Hospital of Stomatology, Sichuan University, Chengdu, Sichuan, China; ^2^Department of Oral and Maxillofacial Surgery, Southwest Hospital, Army Medical University, Chongqing, China; ^3^School of Nursing, Army Medical University, Chongqing, China; ^4^State Key Laboratory of Trauma, Burn and Combined Injury, Third Military Medical University, Chongqing, China; ^5^Department of Stomatology, Daping Hospital, Army Medical University, Chongqing, China

**Keywords:** *Streptococcus vestibularis*, temperature phage, lysogeny, oral phage, Siphoviridae family, prophage engineering

## Abstract

**Introduction:**

Bacteriophages play a vital role in the human oral microbiome, yet their precise impact on bacterial physiology and microbial communities remains relatively understudied due to the limited isolation and characterization of oral phages. To address this gap, the current study aimed to isolate and characterize novel oral phages.

**Methods:**

To achieve this, oral bacteria were isolated using a culture-omics method from 30 samples collected from healthy individuals. These bacteria were then cultured in three different types of media under both aerobic and anaerobic conditions. The samples were subsequently subjected to full-length 16S rRNA gene sequencing for analysis. Subsequently, we performed the isolation of lytic and lysogenic phages targeting all these bacteria.

**Results:**

In the initial step, a total of 75 bacterial strains were successfully isolated, representing 30 species and 9 genera. Among these strains, *Streptococcus* was found to have the highest number of species. Using a full-length 16S rRNA gene similarity threshold of 98.65%, 14 potential novel bacterial species were identified. In the subsequent phase, a temperate phage, which specifically targets the human oral commensal bacterium *S. vestibularis* strain SVE8, was isolated. The genome of *S. vestibularis* SVE8 consists of a 1.96-megabase chromosome, along with a 43,492-base pair prophage designated as SVep1. Annotation of SVep1 revealed the presence of 62 open reading frames (ORFs), with the majority of them associated with phage functions. However, it is worth noting that no plaque formation was observed in *S. vestibularis* SVE8 following lytic induction using mitomycin C. Phage particles were successfully isolated from the supernatant of mitomycin C-treated cultures of *S. vestibularis* SVE8, and examination using transmission electron microscopy confirmed that SVep1 is a siphovirus. Notably, phylogenetic analysis suggested a common ancestral origin between phage SVep1 and the cos-type phages found in *S. thermophilus*.

**Discussion:**

The presence of SVep1 may confer immunity to *S. vestibularis* against infection by related phages and holds potential for being engineered as a genetic tool to regulate oral microbiome homeostasis and oral diseases.

## Introduction

The human microbiome has attracted significant attention from researchers and has become a valuable model system for studying diverse microbial communities (Milligan-McClellan et al., 2022). Interspecies networks within the microbiome can influence energy metabolism pathways and have implications for human health (Cox et al., 2022). Among the various human microbiomes, the gut and oral microbiota exhibit the highest diversity, hosting hundreds of coexisting species, including bacteria and viruses (Szafrański et al., 2021; Cao et al., 2022). However, in the past decade, phages in the gut microbiota have received more attention compared to phages in the oral microbiota, which have been relatively understudied (Federici et al., 2021; Shen et al., 2023).

Bacteriophages are the most prevalent viruses in the human microbiome (Khan Mirzaei and Deng, 2022). They are viruses that infect and replicate within bacterial or archaeal cells, potentially exerting a significant impact on microbial composition and function (Federici et al., 2021). Bacteriophages can either be virulent, causing immediate destruction of bacterial cells after replication, or temperate, integrating their genome with that of the host and participating in horizontal gene transfer, including the transfer of virulence factors (Shalon et al., 2023).

Since the early days of microbiology, extensive research has focused on the bacteria constituting the oral microbiome, with over 70% of these bacteria now being isolated and described (Huang et al., 2023). However, phages within the oral microbiome remain one of the least understood components, primarily due to challenges in detection, isolation, and characterization (Baker et al., 2017; Martínez et al., 2021). To date, only a few phages targeting specific oral bacteria, such as *Actinomyces* spp. (Delisle et al., 2006), *Aggregatibacter* spp. (Resch et al., 2004), *Enterococcus* spp. (Khalifa et al., 2015; Bhardwaj et al., 2020), *Streptococcus* spp. (van der Ploeg, 2008, 2010; Ben-Zaken et al., 2021), *Fusobacterium* spp. (Machuca et al., 2010; Kabwe et al., 2019), *Veillonella* spp. (Hiroki et al., 1976), *Treponema* spp. (Mitchell et al., 2010), and *Lactobacillus casei* (Meyers et al., 1958) has been discovered. Moreover, their potential use for combating oral pathogens and controlling oral biofilms has been discussed and investigated (Szafrański et al., 2017).

While phages have been identified in the oral cavity, our understanding of phages specific to oral streptococci, particularly in comparison to other lactic acid bacteria like *Lactococcus lactis* and *Streptococcus thermophilus*, remains limited (Romero et al., 2020; Hanemaaijer et al., 2021). Among the salivarius group of streptococci, which holds great significance for human health, three genetically related species are prominent: *S. salivarius*, *S. vestibularis*, and *S. thermophilus*. *Streptococcus thermophilus* is primarily utilized in cheese production, while *S. salivarius* and *S. vestibularis* are common commensal organisms that have the potential to cause opportunistic illnesses in humans (Delorme et al., 2015). Notably, *S. thermophilus* has been associated with over 300 virulent and temperate phages to date (Quiberoni et al., 2010; Philippe et al., 2020). Additionally, the temperate phage YMC-2011 of *Streptococcus salivarius* has been isolated and identified (Chou et al., 2017). Recently, a potential prophage from *S. vestibularis* was described in the oral flora of children with autism (Tong et al., 2022). However, no phage from *S. vestibularis* has been isolated and characterized thus far.

Phage communities within the oral microbiome exhibit high biogeographic variation. Like bacterial communities, phage communities are highly unique to each individual (Edlund et al., 2015). Simultaneously, both the oral microbiome and phage community undergo changes during disease progression, and dysbiosis in phage populations may contribute to the development of oral disorders (Abeles and Pride, 2014). Nevertheless, due to the limited number of experimentally described phages and the absence of accessible model systems, the ecological and physiological significance of phages in the oral microbiome and their impact on host–microbe interactions remain unclear (McLean et al., 2016).

In this study, we present the discovery of a novel inducible prophage, SVep1, isolated from *S. vestibularis* strain SVE8 obtained from human saliva. The complete genome sequence of *S. vestibularis* strain SVE8 reveals a 1.96-Mb chromosome with a 43,492-bp prophage. Phylogenetic analysis demonstrates that SVep1 represents a unique lineage of siphovirus.

## Materials and methods

### Sample collection and bacteria isolation

In this study, non-irritating saliva samples were collected from 30 healthy individuals with the approval of the Ethics Committee of the Third Military Medical University (NO: AF/SC-08/1.0). Firstly, the saliva was collected in 50 mL sterile centrifuge tubes. Secondly, the saliva samples were inoculated on brain heart infusion (BHI) agar plates with or without 5% defibrinated sheep blood, as well as on MRS agar plates, for the isolation of individual bacteria. The BHI agar plates and the sheep blood-supplemented BHI agar plates were incubated at 37°C for 48 h in a microaerophilic incubator (5% O_2_, 5% CO_2_, balanced with N_2_), while the MRS agar plates were incubated at 37°C for 48 h in an anaerobic workstation. Next, to obtain different types of organisms, colonies with different shapes, colors, textures, and sizes were selected and resuspended in sterile water, followed by a 1 million-fold dilution. A volume of 100 μL of the diluted bacterial solution was then spread onto the corresponding agar plate surface. Subsequently, the inoculated nutrient agar plates were incubated at 37°C for 48 h in the respective incubators mentioned earlier. Afterwards, a single colony from the second-generation plates was inoculated into a liquid medium for proliferation and preservation. Subsequently, a single colony on the second-generation plate was inoculated to a liquid medium for proliferating and conservation. Finally, the remaining saliva samples were mixed with SM buffer (Parras-Moltó et al., 2018) and stored at 4°C for subsequent isolation of oral phages.

### Identification of bacterial strains by 16S rRNA gene sequencing

Take 1 mL of the bacterial solution and centrifuge it at 12,000 × g for 2 min. Discard the supernatant and wash the pellet three times with sterile normal saline. Add 100 μL of sterile water and heat the suspension in a boiling water bath for 10 min. The resulting bacterial solution was used as a template for amplifying the full-length sequence of the bacterial 16S rRNA gene using universal primers 27F (5′-AGAGTTTGATCCTGGCTCAG-3′) and 1492R (5′-TACGACTTAACCCCAATCGC-3′). The PCR reaction system (50.0 μL) consisted of 2x Es Taq Master Mix (25.0 μL), 1.0 μL of each upstream and downstream primer, 1.0 μL of DNA template, and 22.0 μL of ddH2O. The amplification program consisted of an initial denaturation step at 94°C for 5 min, followed by 30 cycles of denaturation at 94°C for 30 s, annealing at 51°C for 30 s, extension at 72°C for 100 s, and a final extension at 72°C for 7 min. The PCR products were then analyzed by 1% agarose gel electrophoresis. The PCR products exhibiting the target bands were selected and sent to Tsingke Biotechnology Co., Ltd. for sequencing. The obtained 16S rRNA gene sequences were searched and compared using BLAST against the GenBank 16S rRNA gene database.^1^

### Isolation of lytic phage

The isolation of bacteriophages was performed following the previously described method (Shen et al., 2018b). A total of 50 mL of sewage from the Southwest Hospital and the remaining saliva samples stored at 4°C were centrifuged at 10,000 × g for 10 min. The resulting supernatant was then filtered through a 0.45 μm sterile filter, and 100 μL of the filtered supernatant was added to a 96-well plate. Next, logarithmic phase host bacteria were added to the same 96-well plate and cultured overnight at 37°C in a microaerophilic incubator (5% O_2_, 5% CO_2_, balanced with N_2_) or an anaerobic incubator. After incubation, the mixture was centrifuged at 21,000 × g for 1 min, and the supernatant was filtered using a 0.22 μm sterile filter. Subsequently, 10 μL of the filtered supernatant was mixed with 200 μL of the host bacteria in a 15 mL centrifuge tube. To this mixture, 5 mL of brain heart infusion (BHI) agar or De Man, Rogosa, and Sharpe (MRS) soft agar was added. The content was then poured onto the surface of an agar plate. All the plates were incubated overnight at 37°C, and the resulting plaques were detected on the top agar.

### Induction of temperate phage

All the previously isolated bacteria were cultured overnight at 37°C with 5% O_2_ and 5% CO_2_ or in an anaerobic incubator using the aforementioned liquid medium (Chou et al., 2017). The overnight bacterial cultures were then diluted 1:100 with fresh BHI liquid medium. An early exponential-phase culture, with the optical density (OD) values at 600 nm (OD_600_) ranging from 0.25 to 0.3, was treated with mitomycin C at a final concentration of 0.3 μg/mL for a duration of 3–8 h. The clarity of the solution was assessed at the end of the treatment period. Following treatment, cultures that exhibited transparency were subjected to centrifugation at 3,500 rpm for 10 min at 4°C, and the supernatant was carefully collected. To remove any cellular debris present in the supernatant, filtration was performed using a 0.45-μm-pore-size membrane. Subsequently, a method similar to the one employed for isolating lytic phages was used to detect the presence of plaques in lysogenic phages.

### Genome sequencing and assembly of *Streptococcus salivarius*

The genomic DNA was extracted using the SDS method (Wilson, 2001). Subsequently, the extracted DNA was subjected to agarose gel electrophoresis for detection, and its concentration was quantified using a Nanodrop spectrophotometer (Thermo Scientific). To sequence the entire genome of *Streptococcus salivarius*, a combination of the Nanopore PromethION platform and Illumina NovaSeq PE150 sequencing was employed. The sequencing services were provided by Beijing Novogene Bioinformatics Technology Co., Ltd. The obtained sequencing data, including the PE150 data and Nanopore data, were used for assembly. The software tool Unicycler (Wick et al., 2017) was utilized to perform the assembly process. Unicycler integrates the data from both sequencing platforms to generate a comprehensive assembly. Subsequently, the reads were compared to the assembled sequence, and the distribution of sequencing depth was analyzed. Based on the sequence length and alignment, the assembled sequence was evaluated to distinguish between a chromosomal sequence and a plasmid sequence. Additionally, the circularity of the assembled genome was checked.

### Genome component prediction

Genome component prediction included the prediction of the coding gene, repetitive sequences, non-coding RNA, genomics islands, transposon, prophage, and clustered regularly interspaced short palindromic repeat sequences (CRISPR). The available steps proceeded as follows: (1) For bacteria, we used the GeneMarkSprogram (Besemer et al., 2001) to retrieve the related coding gene. (2) The interspersed repetitive sequences were predicted using the RepeatMasker (Saha et al., 2008). The tandem Repeats were analyzed by the TRF (Tandem repeats finder). (3) Transfer RNA (tRNA) genes were predicted by the tRNAscan-SE (Lowe and Eddy, 1997). Ribosome RNA (rRNA) genes were analyzed by the rRNAmmer (Lagesen et al., 2007). Small nuclear RNAs (snRNA) were predicted by BLAST against the Rfam database (Gardner et al., 2009). (4) The IslandPath-DIOMB program (Hsiao et al., 2003) was used to predict the Genomics Islands and transposon PSI was used to predict the transposons based on the homologous blast method. The PHAST (Zhou et al., 2011) was used for the prophage prediction and the CRISPRFinder (Grissa et al., 2007) was used for the CRISPR identification.

### Gene function annotation

Gene functions were assigned using a comprehensive approach. The Gene Ontology database (GO, http://www.geneontology.org/) was utilized to acquire gene function information through IPRscan. The functions were categorized into three groups: cellular component (CC), molecular function (MF), and biological process (BP). To predict genes involved in signaling pathways, a BLAST analysis was performed. Furthermore, the functions of gene-encoded proteins were determined by aligning them with the Cluster of Orthologous Groups of Proteins (COG, https://www.ncbi.nlm.nih.gov/COG/) database. The COG database is specifically designed to infer protein functions based on the evolutionary relationships of proteins encoded by whole genomes. Transporters were annotated using the Transporter Classification Database^2^ via BLAST analysis. This database provides comprehensive information on various transporter systems. For pathogenic bacteria, additional analyses were conducted to assess pathogenicity and drug resistance. The PHI (Pathogen Host Interactions), VFDB (Virulence Factors of Pathogenic Bacteria), and ARDB (Antibiotic Resistance Genes Database) were employed for these analyses. Carbohydrate-Active enzymes were predicted using the Carbohydrate-Active enZYmes Database, which is specifically dedicated to enzymes involved in the breakdown, biosynthesis, and modification of carbohydrates.

### Phylogenetic analysis

To construct the phylogenetic tree of SVE8, a multi-FASTA file containing *coaE* gene sequences was aligned using the ClustalW algorithm within the MEGAX software version 10.0.5. The neighbor-joining (NJ) method was employed for phylogenetic analysis. Gaps and missing data were completely deleted, and a bootstrap test with 1,000 replicates was performed to assess the reliability of the phylogenetic hypothesis. The MEGAX software, developed by Kumar et al. (2018), facilitated these analyses and calculations. Similarly, the phylogenetic tree of SVep1 was constructed using the same methodology as the SVE8 phylogenetic tree. Phylogenetic tree construction and annotation were performed using the iTOL tool.^3^

### Bacterial growth curve of *Streptococcus vestibularis*

Overnight bacterial cultures were diluted at a ratio of 1:100 and inoculated into 96-well plates containing fresh BHI medium. The plates were then placed in a microplate reader, and the OD_600_values were automatically monitored over a period of 12 h. Measurements of the OD_600_ were recorded at 30-min intervals throughout the duration of the experiment. To ensure the reliability of the results, three biological replicates were performed, providing robustness and allowing for the assessment of potential variations.

### Transmission electron microscopy

Phage particles were carefully deposited onto carbon-coated copper grids and allowed to settle for a duration of 10 min. Subsequently, the grids were stained with phosphotungstic acid (PTA [pH 7.0]) for 5 s to enhance contrast. The grids were then examined under a Philips EM 300 electron microscope (Yang et al., 2017), enabling visualization of the phage particles at high magnification. To estimate the sizes of the icosahedral capsid and the tail, five randomly selected images were analyzed using AxioVision LE software. This software facilitated measurements and calculations based on the captured images, providing a means to estimate the sizes of the capsid and tail structures of the phage particles.

### Relative quantification of phage particles

The quantification of phage particles generated after induction with MMC (mitomycin C) was performed using qPCR (quantitative polymerase chain reaction). For this analysis, the lysed supernatant, treated with DNaseI to remove any remaining extracellular DNA, served as the template for the qPCR reactions. The qPCR reactions were carried out using iQ SYBR green supermix (Bio-Rad) and the CFX96 real-time PCR system (Bio-Rad). Specifically designed primers targeting the SVep1 integrase, single-stranded DNA-binding protein, and the 16S rRNA gene of *S. vestibularis* were used for the qPCR reactions. The primer details are provided in Table 1. To determine the copy numbers of the phage particles, the mean quantification cycle (Cq) values of the 16S rRNA gene were compared to the Cq values of the phage-specific genes. The copy numbers were calculated using the formula 2^ΔCq^, which takes into account the difference in Cq values between the target genes (Shen et al., 2018a).

**Table 1:** Primers used in this study.

Gene/protein	Primer	Sequence (5′-3′)
Bacterial 16S rRNA gene	SVE-F5	TGGAGCATGTGGTTTAATTCGA
	SVE-R3	TGCGGGACTTAACCCAACA
Integrase	SVep1-intF	GAGGGCTATTCGTGACGGGATC
	SVep1-intR	GGGAACCAGTTACGCCAATCAGA
Single-stranded DNA-binding protein	SVep1-ssF	CTCGCTTGTTGGTCGTCTCACC
	SVep1-ssR	TGGTCAATGCGCCTTTCTTAGC

### Analysis of the host range for SVep1

The host range of SVep1 was determined through a series of experiments. Initially, strains of interest were cultured overnight. Subsequently, 10 μL of the overnight culture was mixed with 100 μL of the SVep1 prophage and incubated for 30 min to allow for phage adsorption. After incubation, 20 μL of the co-culture was pipetted onto a BHI plate, and single colonies were obtained through streaking. Individual colonies were then selected, cultured overnight, and used for DNA extraction. Genomic DNA (gDNA) was extracted from the overnight bacterial cultures using the TIANamp Bacteria DNA kit. The concentration and quality of the extracted gDNA were assessed using a NanoDrop spectrophotometer. This step ensured that the DNA samples met the required criteria for subsequent analyses. To detect the presence of SVep1-specific genes in the bacterial genome in batches, the templates obtained by the PBC method (Liu et al., 2022) were subjected to PCR (polymerase chain reaction). This involved amplifying the specific gene of interest using primers designed to target SVep1. The presence or absence of the SVep1-specific gene was determined based on the results of the PCR amplification.

## Results

### Isolation and identification of oral bacteria

**Figure 1:**
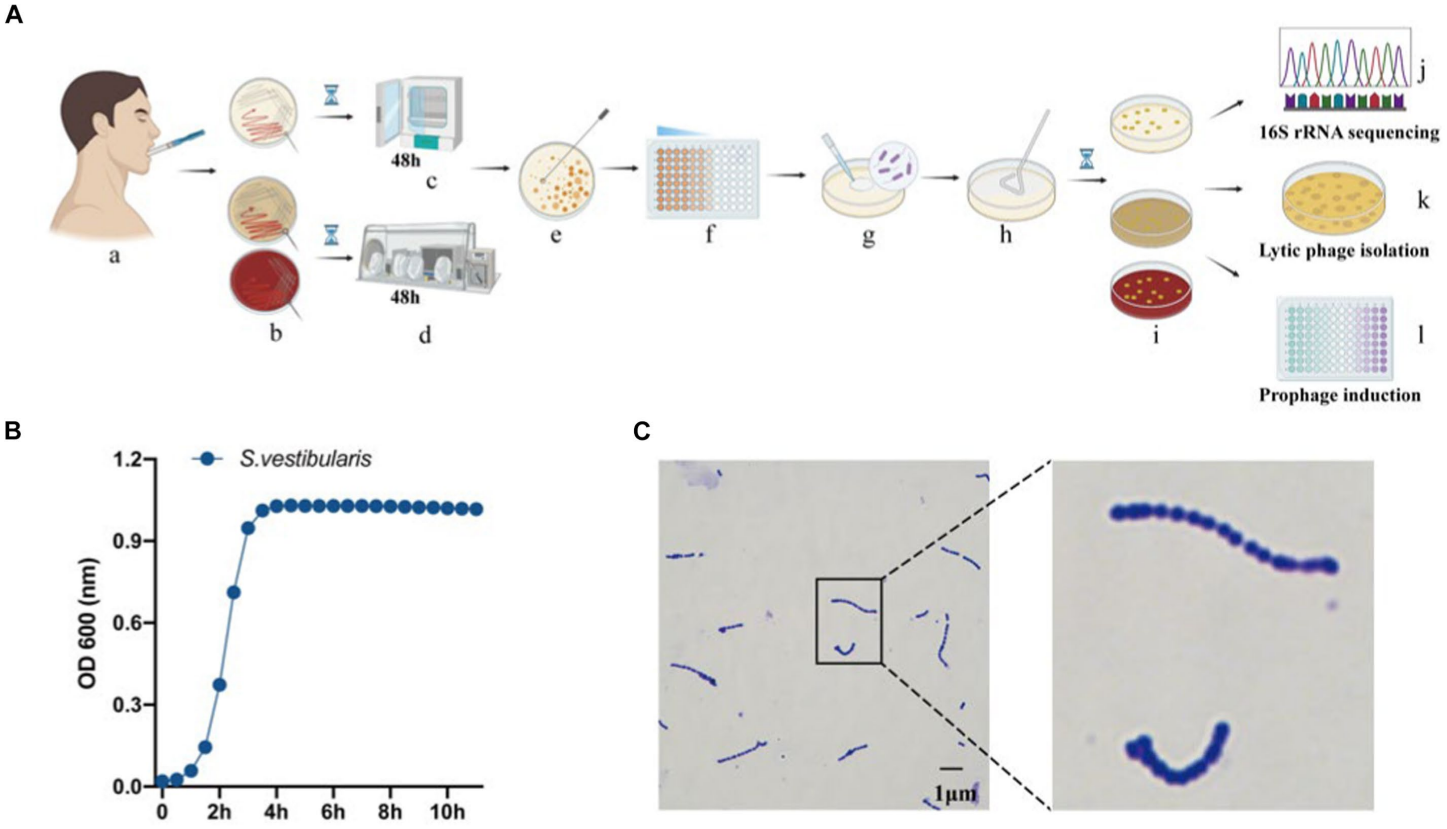
Isolation and identification of oral bacteria and phages. **(A)** The workflow of the study, created using Biorender (https://app.biorender.com). **(a)** Saliva collection. **(b)** Inoculation of saliva on different agar plates, including BHI agar with or without 5% defibrinated sheep blood, and MRS agar. Cultivation of plates in a microaerophilic incubator **(c)** and anaerobic workstation **(d)**. **(e)** Selection of a single colony. **(f)** Dilution of the selected colony. **(g,h)** Spreading of the diluted culture. **(i)** Incubation of the cultures. **(j)** Sequencing of the 16S rRNA gene from pure bacterial cultures. **(k)** Isolation of lytic phages. **(l)** Isolation of temperate phages. **(B)** Measurement of optical density (OD_600_) of bacterial cultures for *Streptococcus vestibularis.* **(C)** Gram stain of *S. vestibularis.*

In this study, we conducted full-length sequencing of the 16S rRNA gene for all isolated bacteria (Figure 1A). The results revealed a diverse range of species, with a total of 30 different species, 9 genera, and 5 phyla, namely Firmicutes, Bacteroidetes, Actinobacteria, Fusobacteria, and Proteobacteria. Among them, the phylum Firmicutes exhibited the highest species diversity, accounting for 89.3% of the total number of bacterial strains. Actinobacteria accounted for 5.3% of the strains, making it the second most abundant phylum. It is worth noting that this analysis, based on a threshold of <98.65% homology in the alignment of the 16S rRNA gene, identified 14 potential novel species (Kim and Yu, 2014). According to the data presented in Table 2, the dominant streptococcal species in human saliva were S. orals and *S. salivarius*, with 21 and 13 strains isolated, respectively. These findings indicate that S. orals and *S. salivarius* are the most prevalent streptococcal species in the oral microbiome.

**Table 2:** Bacteria isolated from saliva in 30 healthy subjects and phage isolated in this work.

Phylum	Genus (frequency)	Strain	Number	Lytic phage isolated	Prophage induction
*Firmicutes*	*Streptococcus (62)*	*Streptococcus vestibularis*	2	-	1
		*Streptococcus gwangjuense*	2	-	-
		*Streptococcus ilei*	1	-	-
		*Streptococcus infantis*	1	-	-
		*Streptococcus intermedius*	1	-	-
		*Streptococcus mitis*	2	-	-
		*Streptococcus oralis*	21	-	-
		*Streptococcus parasanguinis*	2	-	-
		*Streptococcus pneumoniae*	1	-	-
		*Streptococcus pseudopneumoniae*	1	-	-
		*Streptococcus rubneri*	1	-	-
		*Streptococcus salivarius*	13	-	-
		*Streptococcus sanguinis*	1	-	-
		*Streptococcus shenyangsis*	2	-	-
		*Streptococcus symci*	1	-	-
		*Streptococcus thermophilus*	3	-	-
		*Streptococcus timonensis*	3	-	-
		*Streptococcus toyakuensis*	1	-	-
		*Streptococcus cristatus*	1	-	-
		*Streptococcus vulneris*	2	-	-
	*Granulicatella (1)*	*Granulicatella adiacens*	1	-	-
	*Gemella (4)*	*Gemella haemolysans*	1	-	-
		*Gemella taiwanensis*	3	-	-
*Bacteroidetes*	*Prevotella (2)*	*Prevotella jejuni*	1	-	-
		*Prevotella melaninogenica*	1	-	-
*Actinobacteria*	*Actinomyces (1)*	*Actinomyces graevenitzii*	1	-	-
	*Schaalia (2)*	*Schaalia odontolytica*	2	-	-
	*Cutibacterium (1)*	*Cutibacterium acnes*	1	-	-
*Fusobacteria*	*Fusobacterium (1)*	*Fusobacterium periodonticum*	1	-	-
*Proteobacteria*	*Neisseria (1)*	*Neisseria sicca*	1	-	-
Total			75	-	1

Subsequently, we conducted lytic and lysogenic phage isolation experiments on all 75 bacterial strains. Regrettably, despite our efforts, we were unable to isolate any lytic phages from the sewage and saliva samples, as no plaques were observed on the experimental plates. However, during the induction of lysogenic phages using mitomycin C, we made an interesting observation. One particular strain of bacteria underwent a significant change in optical clarity at the conclusion of the experiment. To further confirm the identity of this strain, we performed a Gram stain, and the results are depicted in Figure 1C. The stained cells exhibited the characteristic gram-positive nature and a chain-like arrangement, which, when combined with the results of the 16S rRNA sequencing (Figure 1C), led us to the conclusion that this strain of bacteria is indeed *S. vestibularis*. Furthermore, we monitored the growth status of this strain of bacteria (Figure 1B), and it reached the logarithmic phase at 2 h, which provides guidance for subsequent exploration of prophage induction conditions.

### General genomic features of *Streptococcus vestibularis* SVE8

**Figure 2:**
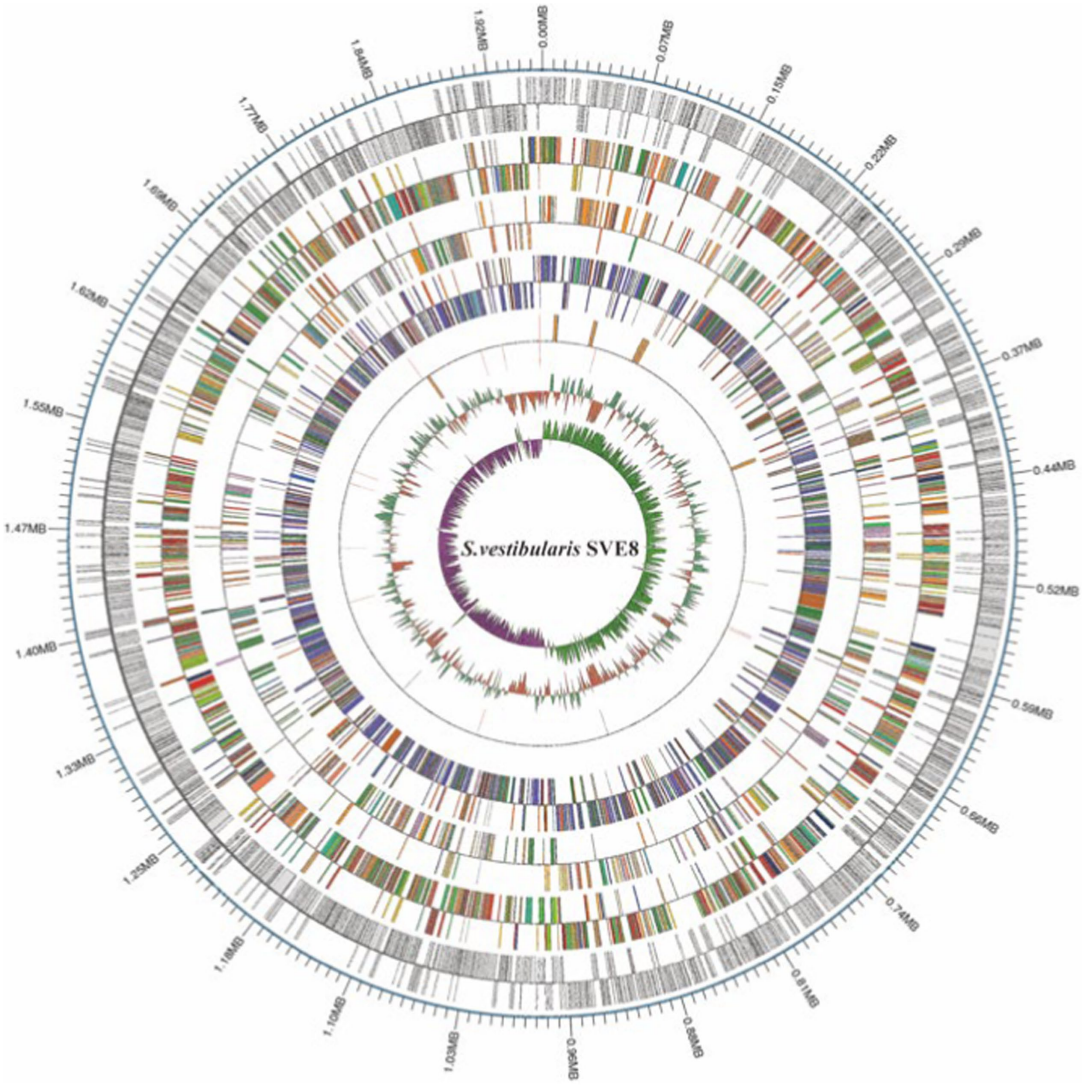
The circular map of the SVE8 chromosome. The 13 concentric circles represent the following (from outermost to innermost): Circle 1: DNA base position. Circles 2 and 3: Protein-coding genes on the forward and reverse strands, respectively. Circles 4 and 5: COG (Cluster of Orthologous Groups) functional classification of protein-coding genes on the forward and reverse strands, respectively. Circles 6 and 7: KEGG (Kyoto Encyclopedia of Genes and Genomes) functional classification of protein-coding genes on the forward and reverse strands, respectively. Circles 8 and 9: GO (Gene Ontology) functional classification of protein-coding genes on the forward and reverse strands, respectively. Circles 10 and 11: rRNA (5S, 16S, and 23S), tRNA, and sRNA genes on the forward and reverse strands, respectively. Circle 12: Relative G + C content, where green (outward) and red (inward) represent values higher and lower than the average of 50.63%, respectively. Circle 13: GC skew ([G − C]/[G + C]), where lime (outward) and magenta (inward) indicate positive and negative values, respectively, representing the leading and lagging strands.

**Figure 3:**
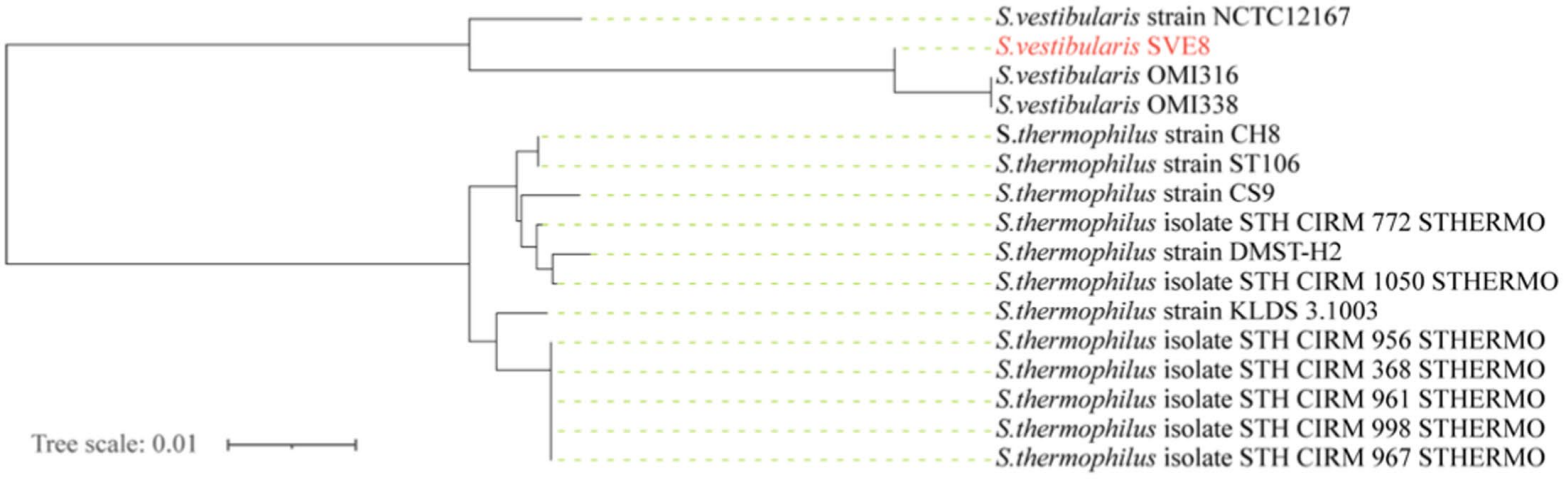
Genome phylogenetic tree based on the housekeeping gene dephospho-coenzyme A kinase (*coaE*) of SVE8 and some Streptococcus strains compared in the study.

We proceeded to sequence the complete genome of *S. vestibularis* strain SVE8, which has the ability to induce lysogenic phages. The main characteristics of the SVE8 genome are illustrated in Figure 2. The genome consists of a circular chromosome spanning 1,952,087 base pairs (bp), with an average GC content of 39.68%. Within the chromosome, we identified 1945 protein-coding genes, 6 rRNA genes, 68 tRNA genes, 3 sRNAs, 30 interspersed repeated sequences, 73 tandem repeats, 45 minisatellite DNAs, 8 genomic islands, 4 prophages, and 1 credible CRISPR loci. A phylogenetic tree based on the housekeeping gene *coaE* of SVE8 was constructed and is presented in Figure 3. Previous studies have reported a close similarity between *S. vestibularis* and *S. salivarius* based on phylogenetic analysis of the 16S rRNA gene sequence (Pombert et al., 2009). Here, we employed the *coaE* (Abdelbary et al., 2021) gene-based phylogenetic tree to demonstrate a closer relationship between *S. vestibularis* and *S. thermophilus.*

### Genome annotation

**Figure 4:**
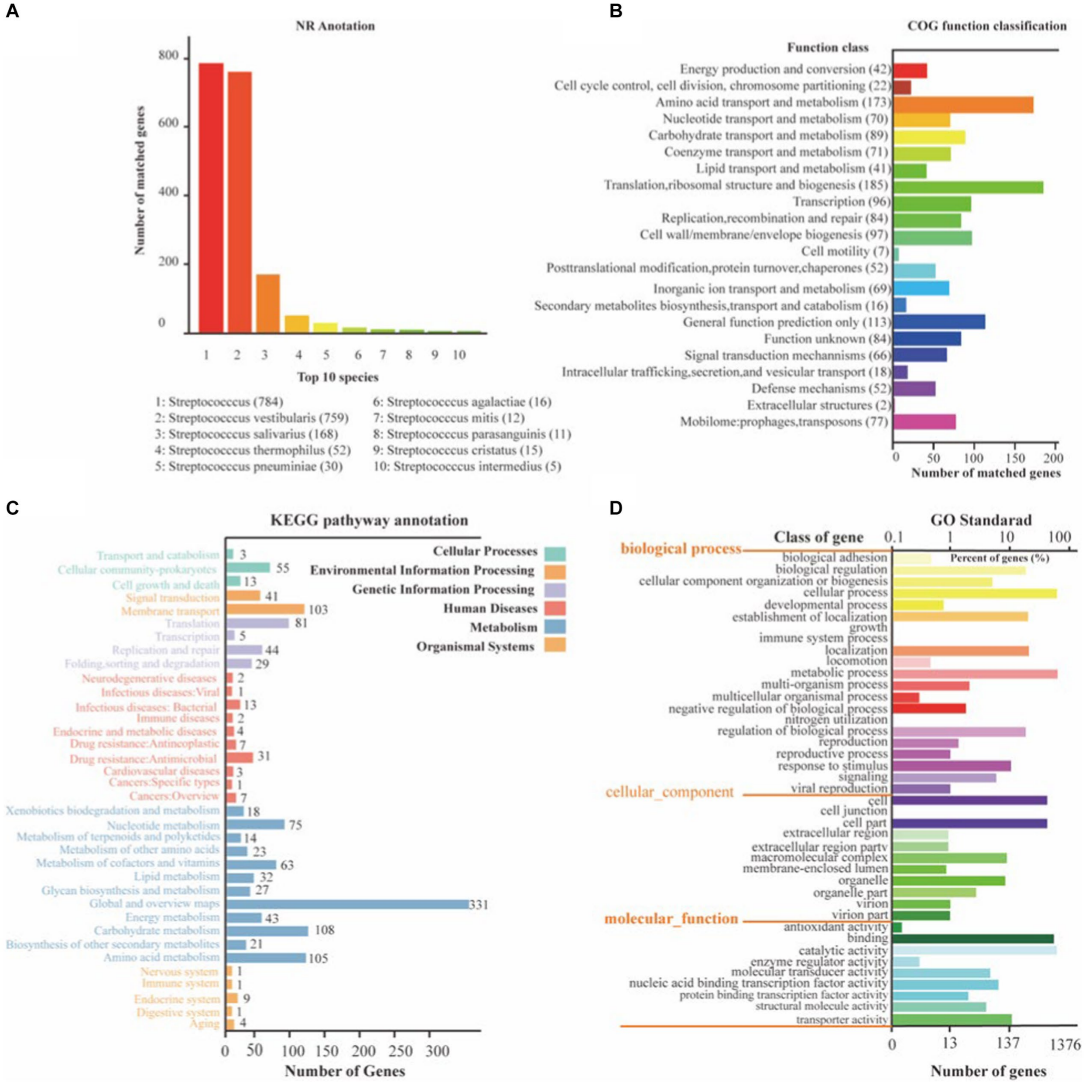
The NR, KEGG, COG, and GO functional annotation of identified genes in SVE8. **(A)** Nr database functional annotation results; **(B)** COG pathway annotation results; **(C)** KEGG pathway annotation results; **(D)** GO functional annotation results.

To gain a comprehensive understanding of the genetic information encoded in SVE8, we performed functional annotation of its genome using various databases, including NR, COG, KEGG, and GO (Figure 4). Through general gene annotation, we identified a total of 1,945 genes, distributed across different databases as follows: 1,886 genes in the NR database, 1,819 genes in the KEGG database, 1,385 genes in the COG database, 180 genes in the TCDB, and 1,375 genes in the GO database. Notably, the top three species represented in the NR database were *S. vestibularis, S. salivarius*, and *S. thermophilus*, reflecting their close phylogenetic relationship (Figure 4A).

Protein functional annotation analysis based on the COG database revealed that the identified genes were primarily associated with translation, ribosomal structure, and biogenesis (185 genes), amino acid transport and metabolism (173 genes), and general function prediction only (423 genes; Figure 4B). It is worth mentioning that 77 genes were annotated to mobilome elements, such as prophages and transposons, suggesting the integration of lysogenic phages within the bacterial genome.

Moreover, KEGG annotation demonstrated that the identified genes were involved in various pathways related to cellular processes, environmental information processing, genetic information processing, human diseases, metabolism, and organismal systems (Figure 4C). GO functional analysis categorized the genes into three ontologies: molecular function (MF) with 1,813 genes, cellular component (CC) with 1,432 genes, and biological process (BP) with 3,059 genes (Figure 4D).

**Figure 5:**
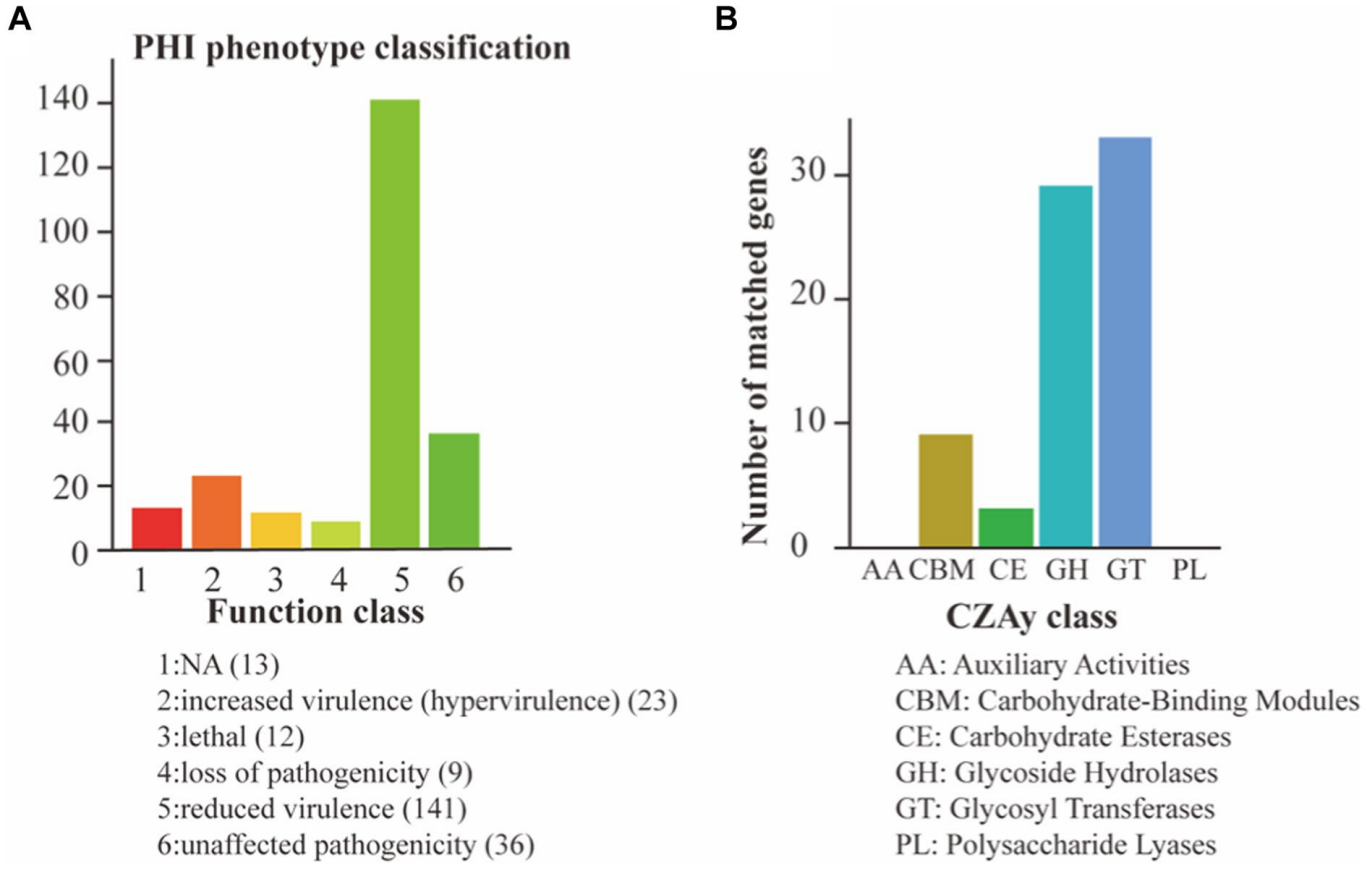
Pathogen analysis results. The abscissa indicates the type of phenotypic mutation and the ordinate indicates the number of genes in the annotation. **(A)** Annotation based on the PHI database; **(B)** Annotation using the CAZy Database.

To assess the pathogenic potential of *S. vestibularis* strain SVE8, we conducted annotations based on the CAZy and PHI databases. In the PHI analysis, a total of 234 genes were clustered into different categories: reduced virulence (141 genes), unaffected pathogenicity (36 genes), increased virulence (23 genes), loss of pathogenicity (9 genes), and lethal (12 genes; Figure 5A). The interactions between pathogen virulence factors and host cell recognition and response mechanisms are complex and constantly evolving, playing a crucial role in the development of infectious diseases (Urban et al., 2020). Interestingly, the genes associated with weakened, lost, or no effect on pathogenicity accounted for more than 50% (79.5%, 186/234) of all annotated genes. This indicates that *S. vestibularis* strain SVE8 likely lacks apparent virulence factors during infection and colonization of hosts.

In the CAZy annotation, we found that 29 genes were enriched in glycoside hydrolases (GHs), 33 genes in glycosyltransferases (GTs), 9 genes in carbohydrate-binding modules (CBM), and 3 genes in carbohydrate esterases (CEs; Figure 5B). These annotations suggest the presence of genes involved in carbohydrate metabolism and potentially related to the utilization of complex carbohydrates by *S. vestibularis* SVE8.

### Induction and observation of *Streptococcus vestibularis* phage SVep1

**Figure 6:**
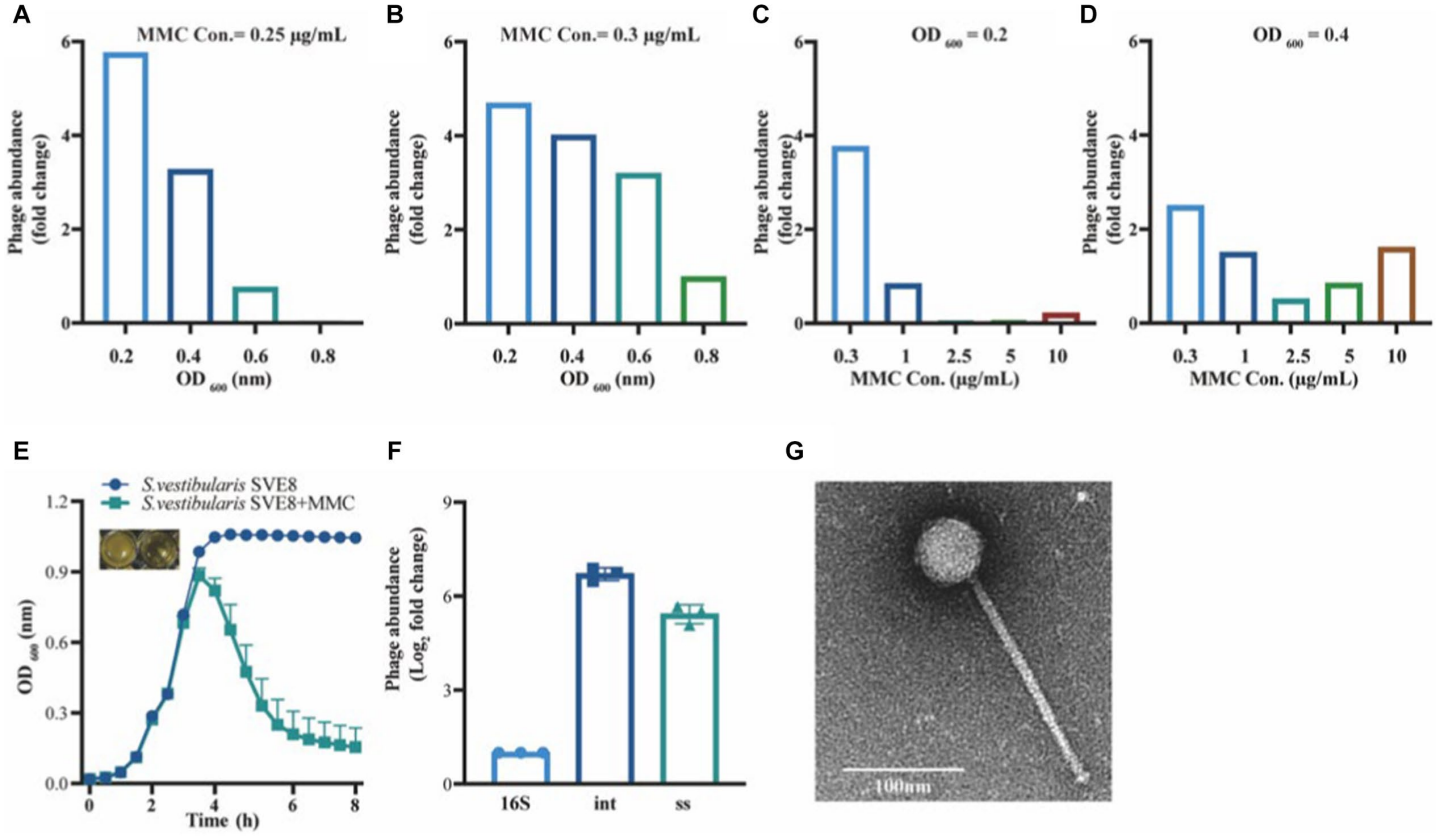
Induction and observation of *Streptococcus vestibularis* phage SVep1. The number of SVep1 particles induced by different MMC concentrations and different OD values was estimated by qPCR. **(A)** Relative quantification of released phage in different bacterial growth states with the addition of 0.25 μg/mL MMC. **(B)** Relative quantification of phage release by 0.3 μg/mL MMC in bacteria at different growth states. **(C)** Addition of different MMC concentrations (0.3, 1, 2.5, 5, and 10 μg/mL) when the bacterial growth reached an OD value of 0.2. **(D)** Treatment with different MMC concentrations when the bacterial growth reached an OD of 0.4. **(E)** Growth curve profiles observed in SVE8 during chemical induction (MMC) at a concentration of 0.3 μg/mL. **(F)** Estimation of the copy number of SVep1 using qPCR. The relative log2 fold changes of the expression levels of phage genes 16S, int., ss in the induced supernatants were analyzed. 16S, bacterial 16S rRNA gene; Int, integrase; ss, single-stranded DNA-binding protein. The data correspond to the means ± S.D. of three different samples, including three technical replicates. **(G)** Transmission electron microscopy (TEM) image of *S. vestibularis* phage SVep1. The phage was negatively stained with PTA.

To determine the optimal conditions for inducing the lytic pathway of lysogenic phage SVep1, several induction experiments were conducted to assess the effect of bacterial growth status and inducer concentration on phage induction (Figures 6A–D). The results showed that when the OD of bacterial growth reached 0.2–0.3 (not exceeding 0.4), the addition of mitomycin C (MMC) at a concentration of 0.2–0.3 μg/mL efficiently induced the release of lytic phages. Figure 6E illustrates the bacterial growth status of *S. vestibularis* after the addition of MMC. Compared to the untreated control, there was a significant decrease in OD_600_ value 2 h after the addition of MMC, and after 6 h of induction, the OD_600_ value approached zero, accompanied by clarification of the culture. This observation indicates that bacterial lysis results in the release of phages into the supernatant.

To explore the quantity of phages released during induction, quantitative PCR (qPCR) was performed on the supernatants obtained from *S. vestibularis* SVE8 cultures. Specific primers targeting genes encoding SVep1 integrase and single-stranded DNA-binding protein were designed for the qPCR analysis. The results revealed that the copy numbers of the genes encoding integrase and single-stranded DNA-binding protein were 6.71 ± 0.20 fold and 5.42 ± 0.30 fold (Log2 fold-change) higher than that of the SVE8 16S rRNA gene, respectively. This significant increase in gene copy numbers indicates the successful presence of phages in the supernatant (Figure 6F).

Temperate phage particles were induced from SVE8 using MMC and subsequently enriched by precipitation with PEG-8000. The phage particles were then subjected to electron microscopy imaging. It was clearly observed that the phage had a long non-contractile tail and an icosahedral capsid (Figure 6G). The estimated sizes of the capsid and tail were approximately 50 ± 1 nm in diameter and 193 ± 3 nm in length, respectively.

### Sequence analysis of SVep1

**Figure 7:**
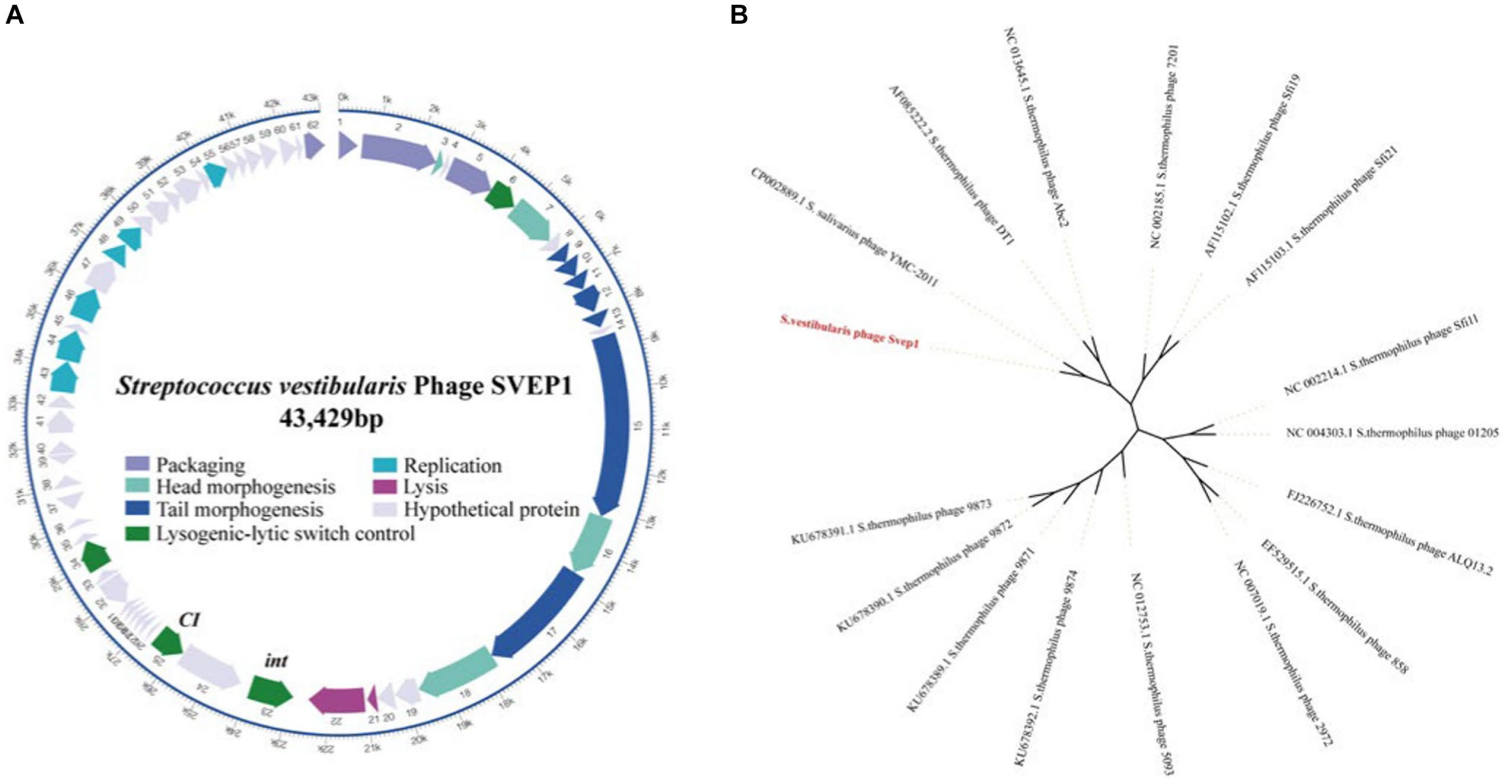
Sequence analysis of SVep1. **(A)** The organization of SVep1 is depicted schematically, indicating the relative location and orientation of each SVep1_phage open reading frame (ORF). ORFs involved in the same pathway for phage production are represented in the same color, while ORFs with unknown functions are shown in gray. The putative origin of replication (ori) and cos site are marked with arrows. **(B)** The phylogenetic relationships of prophage SVEP1 are presented. The phylogenetic tree was constructed using the complete genome sequences and the neighbor-joining method.

The intact prophage SVep1 was identified in the SVE8 genome using the prophage prediction program PHASTER. The SVep1 genome is 43 kb in size with a GC content of 41%, which is similar to its host SVE8, having an overall GC content of 39.68%. SVep1 contains 62 putative open reading frames (ORFs), out of which 28 have been functionally annotated using protein BLAST (Figure 7A). The remaining ORFs show limited homology to sequences in the nucleotide or amino acid databases. The annotated proteins can be classified into several groups, including packaging (terminase), structural proteins (head and tail proteins), transcriptional regulators of the lysogenic-lytic switch (repressor and integrase), DNA replication, and host cell lysis (phage lysin).

To further analyze the phylogenetic relationship of SVep1, its genome sequence was compared to the nucleotide database at the National Center for Biotechnology Information (NCBI). Based on the BLAST results, 15 *S. thermophilus* phages and an *S. salivarius* phage YMC-2011 were selected for phylogenetic analysis (Figure 7B). As expected, the *cos*-type phages DT1, Abc2, Sfi19, Sfi21, and 7,201 form a cluster, while the *pac*-type phages 2,972, 858, ALQ13.2, Sfi11, and O1205 form another cluster. The newly discovered 987 group (phages 9,871, 9,872, 9,873, and 9,874) belongs to a distinct cluster. SVEP and YMC-2011 show close relatedness to cos-type *S. thermophilus* phages, suggesting a common ancestry between them.

### Host range of SVep1

To determine the infectivity of SVep1 toward the salivarius group of streptococci bacteria isolated in this study, we performed PCR to verify the presence of specific phage fragments in the genomes of infected bacteria (Table 3). Unfortunately, no strains among the tested salivarius group bacteria were found to be susceptible to SVep1 infection. Additionally, despite multiple attempts, no plaque were observed when *S. salivarius* and *S. thermophilus* were infected with purified SVep1 phage particles. These findings are consistent with the results obtained for YMC-2011, a temperate phage of *S. salivarius*, which also did not form plaques in the plaque assay and was unable to infect *S. thermophilus* (Chou et al., 2017).

**Table 3:** Host ranges of *Streptococcus vestibularis* bacteriophages on salivaris subgroup.

Genus	Strains	Number of strains used	Sensitive
*Streptococcus*	*Streptococcus vestibularis*	1	-
	*Streptococcus salivarius*	13	
	*Streptococcus thermophilus*	3	

## Discussion

In this study, we initially isolated 75 bacterial strains belonging to 9 genera from 30 saliva samples. Subsequently, we performed the isolation of lytic and lysogenic phages targeting all these bacteria. Fortunately, a lysogenic phage named SVep1 was successfully induced from *Streptococcus vestibularis* SVE8. The presence of SVep1 may confer immunity to *S. vestibularis* against infection by related phages and holds potential for being engineered as a genetic tool to regulate oral microbiome homeostasis and oral diseases.

The field of human microbiota research has experienced a significant resurgence of interest in recent years (Tidjani Alou et al., 2020; Mukhopadhya et al., 2022). While studies on the gut microbiota have gained substantial attention, research on the oral microbiota remains relatively limited (Hou et al., 2022; Schamarek et al., 2023). The oral cavity is known to harbor approximately 700 different bacterial species or phylotypes (Barbour et al., 2022). However, most knowledge about oral bacteria is derived from sequencing studies, and the lack of physical strains greatly hampers subsequent functional investigations (Khelaifia et al., 2023). Several species associated with periodontal diseases (Alves et al., 2022), such as *Porphyromonas gingivalis*, *Tannerella forsythia*, and *Treponema denticola* were not detected in our tested samples. Additionally, we did not find any evidence of commonly implicated tooth decay and dentinal cavity-associated bacteria such as *S. mutans, Lactobacillus*, *Bifidobacterium*, or *Atobobacteria* (Forssten et al., 2010; Caufield et al., 2015; Homayouni Rad et al., 2023).

Due to the limitations of traditional culture methods, the oral bacteria isolated are typically common species in our work. However, researchers have made an interesting discovery by using Yeast extract-casein hydrolysate fatty acids (YCFA) medium and blood culture bottles supplemented with rumen to cultivate bacteria from two stool samples. Within a span of 3 weeks, they successfully isolated 121 different bacteria (Naud et al., 2020). The majority of bacteria in the oral microbiota are anaerobes, as evidenced by studies that have demonstrated a high yield of culture using YCFA medium to cultivate anaerobic bacteria (Browne et al., 2016; Naud et al., 2020). This suggests that the YCFA medium is well-suited for cultivating oral anaerobic bacteria. Thus, it may serve as a valuable tool for future studies aiming to investigate the diversity of the oral microbiota. However, to date, there have been no studies that have fully utilized the YCFA medium to explore the richness and variety of the oral microbiota. In addition to YCFA medium, innovative approaches such as co-culture and reverse genomics have the potential to isolate previously uncultured and fastidious microbes from the oral cavity (Tanaka and Benno, 2015; Cross et al., 2019). By expanding the sampling sites to include areas such as the palate, sublingual region, tongue, and the sides of the teeth, it is possible to obtain a greater diversity of oral bacteria. This comprehensive evaluation of oral bacteria can establish a foundation for the isolation of oral phages and further research in this field.

Based on 16S rRNA genes phylogenetic inferences, the salivarius group comprises three species: *S. salivarius, S. vestibularis*, and *S. thermophilus* (Kawamura et al., 1995). Given their shared physiological traits and habitat, it is reasonable to assume that *S. salivarius* and *S. vestibularis* are more closely related to each other than to *S. thermophilus*. Recent phylogenetic analyses presented in a study strongly support the sister relationship between *S. vestibularis* and *S. thermophilus*, with *S. salivarius* diverging earlier at the base of the salivarius clade (Pombert et al., 2009; Abdelbary et al., 2021). Identifying and typing viridans group streptococci has proven challenging in multiple reports. For example, Matrix Assisted Laser Desorption Ionization/Time Of Flight Mass Spectrometry (MALDI-TOF MS) has demonstrated limitations in accurately identifying streptococci within the salivarius group (Murray, 2010). Similarly, due to the high similarity (>99%) of the 16S rRNA gene among the three species in the salivarius group (Thompson et al., 2013), clear differentiation between them is typically difficult. In our study, we adopted a new typing approach that involves amplification and sequence analysis of the housekeeping gene *coaE* to identify these closely related members of the salivarius group (Abdelbary et al., 2021). Our findings, which align with recent studies, indicate a closer relationship between *S. vestibularis* and *S. thermophilus*. Based on this, we speculate that the phages infecting *S. vestibularis* may also share a closer relationship with those infecting *S. thermophilus*.

While a significant number of gut phages have been isolated and characterized (Shen et al., 2023), the isolation of oral phages remains limited. Despite numerous efforts, the successful isolation of oral bacteriophages has proven challenging. For instance, in a study screening 300 saliva samples from healthy or periodontitis patients and 10 stool samples from colorectal cancer (CRC) patients, only 5 phages were isolated and tested against 11\u00B0*F. nucleatum* bacteria (Wang et al., 2022). Similarly, out of 254 test samples (including saliva, teeth, dental plaque, dental effluent, and general sewage), only one lytic phage targeting *S. mutans*, named SMHBZ8, was identified (Ben-Zaken et al., 2021). Another recent study attempted to isolate lytic phages of *S. mutans* from the saliva of 60 volunteers but did not succeed (Nascimento et al., 2021). It is plausible that the absence of lytic phages in sewage and the 30 saliva samples used in our studies could be attributed to various factors influencing phage isolation. One notable factor is the presence of diverse phage-resistance mechanisms, which may impede the successful isolation of phages (Hampton et al., 2020). To discover novel and unexplored lytic phages, it is essential to develop more sophisticated isolation methods that can overcome these challenges.

Bacteriophages, predominantly long-tailed phages previously classified as Siphoviridae (Turner et al., 2023), constitute the majority of identified viruses in the oral cavity. These phages are believed to play a crucial role in shaping the oral microbiome (Ly et al., 2014). Many of these long-tailed phages exhibit a lysogenic lifestyle, which contributes to the establishment of a dynamic equilibrium within the microbial community and also provides opportunities for the transfer of genetic information among host species (Baker et al., 2017). It is estimated that 60%–70% of known bacterial genomes harbor phages, and as more phages are discovered and sequenced, this percentage is expected to increase (Keen and Dantas, 2018). Although many bacterial genomes contain prophages (Rezaei Javan et al., 2019; Martín-Galiano and García, 2021), the number of lysogenic phages currently isolated from the oral cavity is still limited. It is therefore not surprising that we isolated only one prophage from 75 oral bacterial strains.

Phages typically infect their hosts by binding to specific receptors on the bacterial surface. Although the receptor for phage DT1 that infects *S. thermophilus* has not been identified, it utilizes the phage anti-receptor (Orf18) to attach to the bacterial surface (Duplessis and Moineau, 2001). Phylogenetic analysis reveals that *S. vestibularis* phage SVep1 shares a common ancestor with *S. salivarius* phage YMC-2011 and *S. thermophilus* phage DT1 (Figure 7B), suggesting a potential ability of SVep1 to infect *S. salivarius* and *S. thermophilus*. However, despite multiple attempts to infect the initially isolated *S. thermophilus* and *S. salivarius* strains with purified SVep1 phage particles and observe plaque formation, no successful infections were observed. Furthermore, no plaque formation was observed when SVep1 was used to infect *S. vestibularis* SVE8.

Research on oral phages in the field of dental studies is still in its early stages, and significant advancements are expected in the coming years. Further in-depth research is needed for the isolation of relevant phages and their potential application in the treatment of oral diseases. It is anticipated that with the accumulation of evidence and continued research, phages can be effectively utilized and their limitations can be optimized, leading to their widespread use in the prevention and treatment of oral diseases in humans. As research progresses and expands, it is expected that more types of oral microorganisms will be discovered, additional pathogenic mechanisms will be unveiled, and a greater number of phages will be identified.

In summary, our study has developed a systematic approach for collecting, isolating, and identifying cultured oral microorganisms from a small population, which serves as a valuable resource for studying the oral microbiome. Furthermore, our findings provide a foundation for future investigations on the isolation of oral bacteria and phages. Notably, we successfully isolated a lysogenic phage, SVep1, from *S. vestibularis* obtained from the saliva of healthy volunteers. The isolation of this phage from healthy individuals suggests its potential role in maintaining the oral flora. This discovery prompts further exploration of the potential application of bacteriophage therapy as a promising tool for modulating the oral microbial community.

## Data availability statement

The datasets presented in this study can be found in online repositories. The names of the repository/repositories and accession number (s) can be found at: https://www.ncbi.nlm.nih.gov/, OR238371; https://www.ncbi.nlm.nih.gov/, JAUJGC000000000.

## Ethics statement

The studies involving humans were approved by the Ethics Committee of the Third Military Medical University (NO: AF/SC-08/1.0). The studies were conducted in accordance with the local legislation and institutional requirements. The participants provided their written informed consent to participate in this study.

## Author contributions

MW: Writing – original draft, Investigation, Methodology, Writing – review & editing. YZ: Writing – original draft, Writing – review & editing. YY: Methodology, Writing – review & editing. YG: Methodology, Writing – review & editing. ZC: Data curation, Writing – review & editing. BL: Data curation, Writing – review & editing. YX: Writing – review & editing. XZ: Funding acquisition, Writing – review & editing. YL: Writing – review & editing.

## Funding

The author(s) declare financial support was received for the research, authorship, and/or publication of this article. This work was supported by the Open Project Program of the State Key Laboratory of Trauma, Burn and Combined Injury, Third Military Medical University (SKLKF202108).

## Conflict of interest

The authors declare that the research was conducted in the absence of any commercial or financial relationships that could be construed as a potential conflict of interest.

## Publisher’s note

All claims expressed in this article are solely those of the authors and do not necessarily represent those of their affiliated organizations, or those of the publisher, the editors and the reviewers. Any product that may be evaluated in this article, or claim that may be made by its manufacturer, is not guaranteed or endorsed by the publisher.

## Footnotes

^1^ https://blast.ncbi.nlm.nih.gov/BlastAlign.cgi

^2^ http://www.tcdb.org/

^3^ https://itol.embl.de/
