# Supplementary material for: SVep1, a temperate phage of human oral commensal Streptococcus vestibularis
Source: Front Microbiol. 2023 Sep 12;14:1256669. doi: 10.3389/fmicb.2023.1256669 (PMC10536254; doi:10.3389/fmicb.2023.1256669)
Supplement: Supplementary file 1 [file Table_1.docx]

**Supplementary File**

Table S1. Properties of ORFs of SVep1.

| locus_tag | id | Start | End | Direction | Putative function | Type |
| --- | --- | --- | --- | --- | --- | --- |
| svep1 | svep1_00043 | 33346 | 34125 | + | putative DNA replication protein" | DNA replication and modification |
| svep1 | svep1_00044 | 34137 | 34919 | + | Helicase loader DnaI" | DNA replication and modification |
| svep1 | svep1_00046 | 35233 | 36060 | + | Recombinational DNA repair protein RecT (prophage | DNA replication and modification |
| svep1 | svep1_00048 | 36906 | 37400 | + | Single-stranded DNA-binding protein" | DNA replication and modification |
| svep1 | svep1_00049 | 37416 | 37964 | + | HNH homing endonuclease" | DNA replication and modification |
| svep1 | svep1_00055 | 40065 | 40583 | + | Phage DNA-binding protein" | DNA replication and modification |
| svep1 | svep1_00003 | 2460 | 2651 | + | Phage capsid and scaffold" | Head morphogenesis |
| svep1 | svep1_00007 | 4723 | 5925 | + | Phage capsid protein" | Head morphogenesis |
| svep1 | svep1_00016 | 13224 | 14771 | + | Phage capsid and scaffold" | Head morphogenesis |
| svep1 | svep1_00018 | 17704 | 19704 | + | Phage capsid and scaffold" | Head morphogenesis |
| svep1 | svep1_00021 | 20775 | 21008 | + | Phage holin" | Host lysis |
| svep1 | svep1_00022 | 21075 | 22475 | + | Phage endolysin" | Host lysis |
| svep1 | svep1_00004 | 2674 | 2805 | + | hypothetical protein" | Hypothetical protein |
| svep1 | svep1_00008 | 5930 | 6253 | + | hypothetical protein" | Hypothetical protein |
| svep1 | svep1_00014 | 8467 | 8613 | + | hypothetical protein" | Hypothetical protein |
| svep1 | svep1_00019 | 19717 | 20322 | + | Phage protein (ACLAME 528)" | Hypothetical protein |
| svep1 | svep1_00020 | 20334 | 20771 | + | hypothetical protein" | Hypothetical protein |
| svep1 | svep1_00024 | 24202 | 25794 | - | Phage protein" | Hypothetical protein |
| svep1 | svep1_00026 | 26735 | 26833 | + | hypothetical protein" | Hypothetical protein |
| svep1 | svep1_00027 | 26931 | 27086 | + | hypothetical protein" | Hypothetical protein |
| svep1 | svep1_00028 | 27095 | 27253 | + | hypothetical protein" | Hypothetical protein |
| svep1 | svep1_00029 | 27269 | 27391 | + | gp5" | Hypothetical protein |
| svep1 | svep1_00030 | 27417 | 27566 | + | hypothetical protein" | Hypothetical protein |
| svep1 | svep1_00031 | 27570 | 27719 | + | hypothetical protein" | Hypothetical protein |
| svep1 | svep1_00032 | 27716 | 28429 | - | hypothetical protein" | Hypothetical protein |
| svep1 | svep1_00033 | 28485 | 28712 | + | Phage protein" | Hypothetical protein |
| svep1 | svep1_00035 | 29564 | 29749 | + | hypothetical protein" | Hypothetical protein |
| svep1 | svep1_00036 | 29978 | 30139 | + | hypothetical protein" | Hypothetical protein |
| svep1 | svep1_00037 | 30382 | 30762 | - | hypothetical phage protein" | Hypothetical protein |
| svep1 | svep1_00038 | 30980 | 31270 | + | Phage protein" | Hypothetical protein |
| svep1 | svep1_00039 | 31541 | 31783 | - | hypothetical protein" | Hypothetical protein |
| svep1 | svep1_00040 | 31813 | 32124 | + | hypothetical protein" | Hypothetical protein |
| svep1 | svep1_00041 | 32334 | 32909 | + | hypothetical protein" | Hypothetical protein |
| svep1 | svep1_00042 | 32953 | 33273 | + | hypothetical protein" | Hypothetical protein |
| svep1 | svep1_00045 | 34916 | 35098 | + | Phage protein" | Hypothetical protein |
| svep1 | svep1_00047 | 36053 | 36889 | + | Phage protein" | Hypothetical protein |
| svep1 | svep1_00050 | 37972 | 38364 | + | Phage protein (ACLAME 624)" | Hypothetical protein |
| svep1 | svep1_00051 | 38382 | 38918 | + | hypothetical protein" | Hypothetical protein |
| svep1 | svep1_00052 | 38921 | 39226 | + | hypothetical protein" | Hypothetical protein |
| svep1 | svep1_00053 | 39223 | 39858 | + | ORF26" | Hypothetical protein |
| svep1 | svep1_00054 | 39859 | 40065 | + | hypothetical protein" | Hypothetical protein |
| svep1 | svep1_00056 | 40552 | 40869 | + | Phage protein" | Hypothetical protein |
| svep1 | svep1_00057 | 40866 | 41105 | + | hypothetical protein" | Hypothetical protein |
| svep1 | svep1_00058 | 41111 | 41494 | + | hypothetical protein" | Hypothetical protein |
| svep1 | svep1_00059 | 41494 | 41901 | + | Phage protein" | Hypothetical protein |
| svep1 | svep1_00060 | 41980 | 42390 | + | Phage protein" | Hypothetical protein |
| svep1 | svep1_00061 | 42464 | 42604 | + | hypothetical protein" | Hypothetical protein |
| svep1 | svep1_00006 | 3998 | 4708 | + | Prophage Clp protease-like protein" | Lysogenic-lytic switch control |
| svep1 | svep1_00023 | 22859 | 23932 | - | Phage integrase" | Lysogenic-lytic switch control |
| svep1 | svep1_00025 | 25825 | 26568 | - | CI-like repressor, phage associated" | Lysogenic-lytic switch control |
| svep1 | svep1_00034 | 28808 | 29551 | + | Phage antirepressor protein" | Lysogenic-lytic switch control |
| svep1 | svep1_00001 | 70 | 537 | + | Phage terminase, small subunit" | Packaging and assembling |
| svep1 | svep1_00002 | 581 | 2473 | + | Phage terminase, large subunit" | Packaging and assembling |
| svep1 | svep1_00005 | 2839 | 4011 | + | Phage portal protein" | Packaging and assembling |
| svep1 | svep1_00062 | 42792 | 43325 | + | HNH endonuclease" | Packaging and assembling |
| svep1 | svep1_00009 | 6246 | 6593 | + | Phage head-tail adaptor (ACLAME 461)" | Tail morphogenesis |
| svep1 | svep1_00010 | 6596 | 7018 | + | Phage tail assembly" | Tail morphogenesis |
| svep1 | svep1_00011 | 7018 | 7392 | + | Phage tail assembly protein" | Tail morphogenesis |
| svep1 | svep1_00012 | 7410 | 8018 | + | Phage major tail protein" | Tail morphogenesis |
| svep1 | svep1_00013 | 8072 | 8428 | + | Phage tail assembly protein" | Tail morphogenesis |
| svep1 | svep1_00015 | 8656 | 13221 | + | Phage tail tape measure" | Tail morphogenesis |
| svep1 | svep1_00017 | 14771 | 17704 | + | Phage tail assembly protein" | Tail morphogenesis |
